# Introducing biometry during outreach: improving visual acuity outcomes in Karamoja subregion, Uganda

**Published:** 2025-09-19

**Authors:** Gladys Atto, Susan Niyigena

**Affiliations:** 1 Opthalmologist: Moroto Regional Referral Hospital, Moroto, Uganda.; 2 Incharge: Eye Department, Moroto Regional Referral Hospital, Moroto, Uganda.

**Continuous monitoring and quality improvement improved outcomes and patient satisfaction, thereby increasing the uptake of cataract surgery.**

**Figure:**
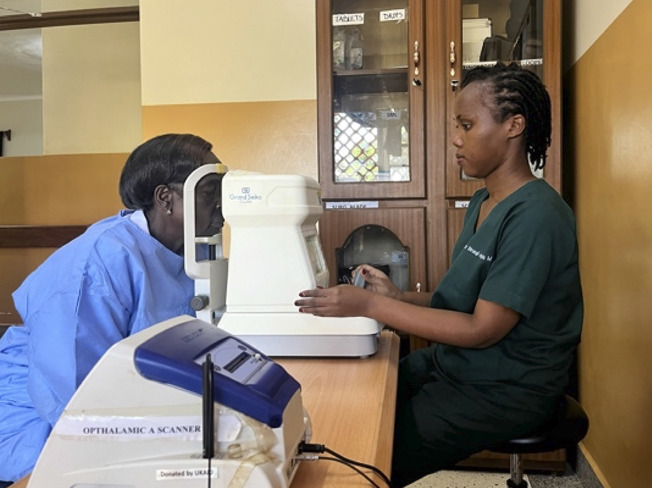
A resident on attachment at Moroto Regional Referral Hospital performs keratometry on a patient using an autorefractor keratometer machine. UGANDA

© GLADYS ATTO CC BY-NC-SA 4.0

Moroto Regional Referral Hospital (MoRRH) Eye Department is a government-owned health facility located in Karamoja subregion in northeastern Uganda. It serves a population of approximately 1.2 million people, sparsely distributed across 27,000 sq km. In 2018, the hospital appointed its first and only ophthalmologist, which enabled it to start offering cataract surgery at the hospital and at three district health facilities during regular outreach visits. Approximately two-thirds (600 to 700 a year) of the department's cataract operations are performed during these outreach visits. One-third (300 to 400 a year) are carried out at the hospital itself.

## Baseline (first) audit

In June 2022, we carried out our baseline audit and realised that only 20% of patients undergoing cataract surgery at the hospital were having biometry. None of those operated on during outreach were undergoing biometry.

Of the patients who did not receive biometry, only 50% had good (6/6 to 6/18) visual acuity on the first postoperative day (see Table 1). At the time of the audit, WHO recommended that – on assessment at 6–8 weeks after surgery – at least 80% of eyes should achieve good visual outcome (6/6–6/18) with available correction, and no more than 5% should have poor outcomes with available correction (<6/60).

**Table 1:** Number and percentage of patients achieving WHO visual acuity cut-offs on first postoperative day, and the percentage for whom the recommended IOL wasn't available

	Good outcome (6/6–6/18)	Borderline outcome (<6/18–6/60)	Poor outcome (<6/60)	Recommended IOL not available
**Percentages recommended by WHO**	> 80%	< 15%	<5%	
Baseline (first) audit (June 2022)	83 (50%)	71 (42.8%)	12 (7.2%)	–
Second audit (December 2022)	122 (48.4%) With pinhole: 177 (70.2%)	103 (40.9%) With pinhole: 49 (19.4%)	27 (10.7) With pinhole: 26 (10.3%)	34%
Third audit (March 2023)	170 (75%) With pinhole: 210 (76%)	50 (22%) With pinhole: 12 (4.3%)	7 (3%) With pinhole: 5 (2%)	25%

“We realised that only 20% of patients undergoing cataract surgery at the hospital were having biometry. None of those operated on during outreach were undergoing biometry.”

Several reasons were given for not performing biometry: the patient volume was high during outreach (approximately 20 per day, or 100 per site visit), so there wasn't enough time; the staff lacked skills; and the equipment was difficult to use.

## First round of quality improvement

We embarked on a quality improvement project with an ambitious target of doing biometry on all cataract patients undergoing surgery by December 2022. We based our project on the quality improvement model adopted from Uganda's National Quality Improvement Framework and Strategic Plan 2021–2025^.1^

We used a Pareto graph (a special bar graph that displays the factors contributing to a problem or outcome) to identify the most frequent issues hindering biometry (equipment and human resource training) and solve these first. We also improved our systems and procedures.

### Equipment

Initially, we tried using a handheld Suoer keratometer (model SW-100) which uses three AA size batteries, and the manual Javal Schiøtz keratometer (YZ-38 keratometer), but it took a lot of time to get the K readings with both machines. This is because most patients present with mature cataracts: getting them to focus takes a lot of time, which delays the biometry process and ultimately the start of surgery.

We therefore started using the Grand Seiko autorefractor keratometry machine (model GR-3100K), which is fairly bulky and requires a table surface and an electric power connection, but is easy to use. This has been giving us quicker readings in an outreach setting. During transportation, it is securely packed in its box.

For axial length measurement, we use the PAC scan (contact), setting it to take an average of ten readings. We have an ophthalmic equipment technician (employed by the hospital) who services the equipment quarterly as part of the routine hospital equipment management; the unit does not incur any additional maintenance costs.

### Human resource training

We trained five of the existing medical personnel in the eye unit (three ophthalmic clinical officers and two ophthalmic nurses) to carry out biometry. The training was a routine part of our department's continuous medical education, so we did not incur any costs nor award certificates to the trainees.

### Systems and procedures

After the training, we designed a **standard operating procedure** that includes the steps for carrying out biometry and guidelines on when to use the different formulae for calculating IOL power, based on the axial length ranges.

We also designed a **checklist** for monthly monitoring, which allows us to check that informed consent is obtained, that equipment calibration is done at the beginning of the procedure, and that the probe position and eye fixation are both correct, with minimal pressure on the cornea.

We rotate personnel to do biometry at the different outreach clinics so that they do not forget their skills. Initially, performing biometry seemed like an additional task to them, but they embraced it in time.

## Second audit

Six months later, in December 2022, we carried out a second audit of the first postoperative day visual acuity outcomes in 252 eyes from three outreach sites.

The results were still below the WHO recommendations: 122 (48.4%) had good outcomes (VA of 6/6 to 6/18), 103 (40.9%) had borderline outcomes (VA of 6/18 to 6/60), and 27 (10.7%) had poor outcomes (VA <6/60).^2^ However, the outcomes improved with pinhole: 177 (70.2%), 49 (19.4%), and 26 (10.3%) had good, borderline, and poor outcomes, respectively (see Table 1).

Of the 252 eyes, 86 (34.1%) did not receive the biometry-calculated IOL power because it was unavailable, which might explain the significant drop in the proportion of borderline outcomes from 40.9% to 19.4% on pinhole assessment. We looked at the results together and identified areas for improvement: inaccurate readings, poor patient selection, limited ranges of IOL powers, and surgical complications.

## Second round of improvement

Personnel received refresher training in biometry. We improved case selection and ordered the various ranges of IOL powers required, with support from Sightsavers. The surgeon shifted from doing can-opener capsulotomy to capsulorrhexis with trypan blue, which greatly reduced the risk of tagging on the anterior capsule flaps; this had been the major cause of posterior capsular tears in 20 (74.2%) of the patients with poor outcomes. From the audit, we also found that, contrary to the standard IOL powers of 21.0 and 22.0 dioptres (D) that we were accustomed to using, 60% of our patients required IOL powers of 23.0 D and above.

## Third audit

Three months later, our audit of 227 eyes (not published) showed remarkable improvement, with 170 (75%), 50 (22%), and 07 (3%) eyes having good, borderline, and poor outcomes, respectively, on the first postoperative day. Although we tried to have various powers of IOL available, 25% of the patients still did not receive the biometry-calculated IOLs because these were out of stock. We assumed this contributed to the bigger proportion of borderline outcomes, since their visual acuity improved with pinhole: 210 (76%), 12 (4.3%) and 5 (2%), respectively, for good, borderline, and poor outcomes. Most of the poor outcomes this time were expected and were due to corneal opacification identified preoperatively. Of the 75% who received the correct IOLs (as specified by the biometry results), 173 (83.6%) had good outcome, while 34 (16.4%) had borderline outcomes. With pinhole, this improved to 193 (93.2%) and 14 (6.8%), respectively. We have not yet achieved the recommended WHO standards, but we believe we are on the right track in improving biometry and visual outcomes (Table 1).

## Patient experience and satisfaction – and the impact on uptake

Applanation A-scan is an uncomfortable but painless procedure. Most of the patients embraced the procedure after explanation and consented, except a few who hesitated at first.

Because of the improved outcomes, we have seen an increase in the uptake of cataract surgery, evidenced by the increased number of walk-ins. We are now able to perform 10–15 cataract operations per week, which is a significant increase.

The first patient satisfaction survey carried out by the hospital this year scored the eye department at 67% satisfaction level. The main cause of dissatisfaction was the long waiting time before surgery (approximately two hours). We are working on improving that as a department by recruiting more staff members to carry out preoperative assessments in less time (we already have a standard operating procedure in place for postoperative assessment). We also plan to start a quality improvement project that we hope will improve the entire cataract service delivery pathway, from the primary eye care facilities to the secondary facility.

### Improving access to IOL power

We are addressing the challenge of limited intraocular lens (IOL) powers through strategic partnerships, inventory management, and sustainability initiatives. Sightsavers Uganda is actively partnering with us to facilitate the bulk purchase of IOLs, ensuring a steady supply. Our ophthalmic nurses use stock cards to manage inventory efficiently. Additionally, as part of our long-term sustainability strategy, we plan to establish a wing for private patients: funds generated will be reinvested in purchasing IOLs. Finally, Uganda's Ministry of Health has included IOLs in the essential supply list of the National Medical Stores, which oversees medical distribution to government health facilities. While this policy has not yet been fully implemented, we continue to engage with stakeholders to expedite its execution.

## Conclusion

Introducing routine biometry in our hospital and outreach cataract surgical services increased the proportion of ‘good’ visual acuity outcomes (6/18 or better presenting vision).

Biometry, when done accurately, can greatly improve visual acuity outcomes and it should be mandatory for all patients undergoing cataract surgery, including in outreach settings. There is need for good, cost-effective, easy-to-use equipment; adequate skilled human resources; and a variable range of IOL powers to improve biometry and postoperative visual outcomes and patient satisfaction.

**Figure:**
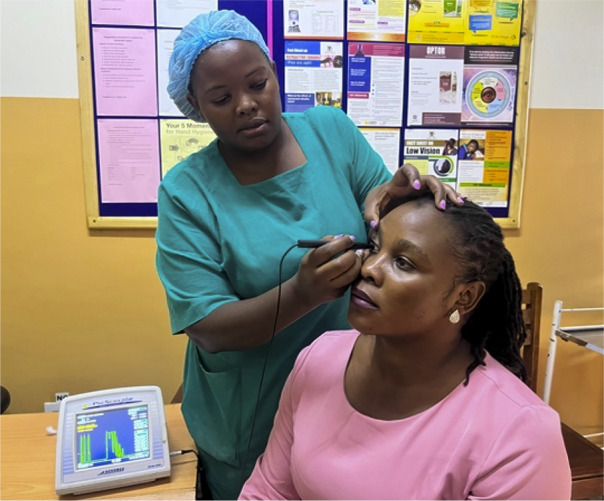
An ophthalmic nurse at Moroto Regional Referral Hospital performs an A-scan. UGANDA

© GLADYS ATTO CC BY-NC-SA 4.0

After starting this biometry and quality improvement process (auditing our results and developing plans for improvement), the proportion of ‘good’ outcomes increased from 48% to 75%, demonstrating the importance of a continuous monitoring and quality improvement process.

**Note**

Patients with corneal opacity often cannot achieve good outcomes, but it is usually still the right choice to remove their cataracts as they could achieve 6/60 or 6/36. This might be a poor or borderline outcome, and therefore affects the audit, but is still a very happy outcome for the patient – especially if they started out with hand movements (HM) or perception of light (PL). In populations with a lot of corneal scarring, or those with a lot of macular disease, the WHO benchmarks may be difficult to reach, but surgeons should not deny surgery to patients with corneal opacity just to improve their chances of hitting a benchmark.

**NEW Research Skills in Global Eye Health: A 16-week Online Course**

**Figure:**
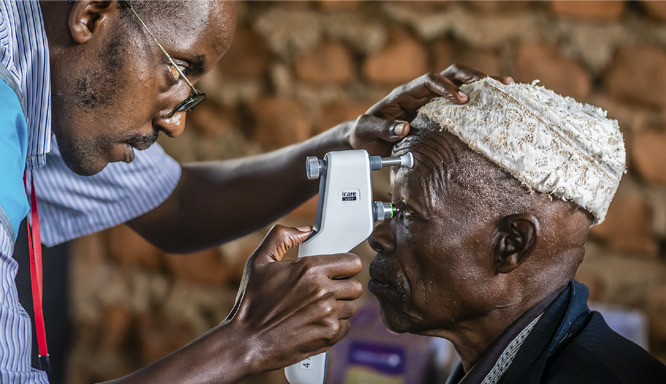

**Start Date:** October 2025

**Institution:** London School of Hygiene & Tropical Medicine (LSHTM)

**Delivery Mode:** Online, flexible learning with expert-led sessions

This short course is designed for eye health professionals and public health practitioners seeking to deepen their understanding of:

- Global challenges in eye health
- Epidemiology and statistics applied to eye care
- Research methods, ethics, and data analysis
- Health economics, planning, and service implementation

**Course Structure:** 4 modules ove 16 weeks + additional 4 weeks for final assessment submission. Includes video lectures, quizzes, discussion forums, and live expert sessions

**Assessment:** End-of-module MCQs, a short video presentation, and a written report

**Figure:**
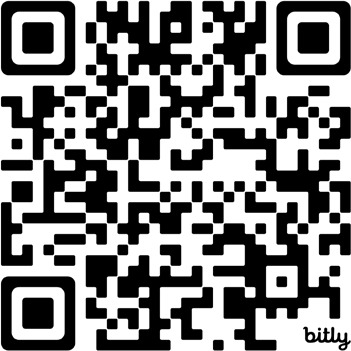
Register your interest:
